# Supplementary material for: Hot spot prediction in protein-protein interactions by an ensemble system
Source: BMC Syst Biol. 2018 Dec 31;12(Suppl 9):132. doi: 10.1186/s12918-018-0665-8 (PMC6311905; doi:10.1186/s12918-018-0665-8)
Supplement: Supplementary file 2 — All relevant MATLAB code. A simple Matlab implement of our predictor. (ZIP 19,742 kb) [file 12918_2018_665_MOESM2_ESM.zip › Additional file 2/Read ASA/ASA website/ASEdb/1A4YB.htm]

 NetSurfP - Protein Surface Accessibility and Secondary Structure Predictions

  

|  |  |  |
| --- | --- | --- |
|  |  | NetSurfP - Protein Surface Accessibility and Secondary Structure Predictions  Technical University of Denmark |

  


---

```
# For publication of results, please cite:
# A generic method for assignment of reliability scores applied to solvent accessibility predictions.
# Bent Petersen, Thomas Nordahl Petersen, Pernille Andersen, Morten Nielsen and Claus Lundegaard
# BMC Structural Biology 2009, 9:51 doi:10.1186/1472-6807-9-51
#
# Column 1: Class assignment - B for buried or E for Exposed - Threshold: 25% exposure, but not based on RSA
# Column 2: Amino acid
# Column 3: Sequence name
# Column 4: Amino acid number
# Column 5: Relative Surface Accessibility - RSA
# Column 6: Absolute Surface Accessibility
# Column 7: Z-fit score for RSA prediction
# Column 8: Probability for Alpha-Helix
# Column 9: Probability for Beta-strand
# Column 10: Probability for Coil
E Q  1A4YB                  1    0.897 160.115   1.014   0.003   0.003   0.994
E D  1A4YB                  2    0.754 108.695  -0.661   0.058   0.017   0.925
E N  1A4YB                  3    0.551  80.710  -1.882   0.109   0.005   0.886
E S  1A4YB                  4    0.476  55.846   0.111   0.246   0.004   0.750
E R  1A4YB                  5    0.389  89.035   0.726   0.858   0.002   0.139
B Y  1A4YB                  6    0.213  45.604  -0.436   0.858   0.002   0.139
E T  1A4YB                  7    0.457  63.372   1.183   0.879   0.010   0.111
E H  1A4YB                  8    0.388  70.523   1.429   0.879   0.010   0.111
B F  1A4YB                  9    0.063  12.664   0.086   0.879   0.010   0.111
B L  1A4YB                 10    0.273  50.041   0.558   0.802   0.014   0.185
E T  1A4YB                 11    0.488  67.658   0.777   0.831   0.044   0.125
E Q  1A4YB                 12    0.287  51.294   1.316   0.605   0.105   0.290
B H  1A4YB                 13    0.187  33.924   0.261   0.199   0.152   0.649
B Y  1A4YB                 14    0.136  29.085   0.112   0.019   0.141   0.840
E D  1A4YB                 15    0.419  60.335   0.200   0.018   0.088   0.893
E A  1A4YB                 16    0.292  32.222  -0.944   0.018   0.019   0.964
E K  1A4YB                 17    0.670 137.881  -0.242   0.018   0.019   0.964
E P  1A4YB                 18    0.551  78.116  -1.303   0.018   0.019   0.964
E Q  1A4YB                 19    0.491  87.710  -1.370   0.018   0.019   0.964
E G  1A4YB                 20    0.601  47.314  -3.212   0.016   0.005   0.979
E R  1A4YB                 21    0.416  95.287  -1.536   0.016   0.005   0.979
E D  1A4YB                 22    0.448  64.571  -1.065   0.053   0.005   0.942
E D  1A4YB                 23    0.520  74.990  -0.854   0.406   0.004   0.590
E R  1A4YB                 24    0.506 115.966   0.518   0.522   0.016   0.462
B Y  1A4YB                 25    0.193  41.180   0.674   0.717   0.014   0.269
B C  1A4YB                 26    0.019   2.724   1.014   0.782   0.003   0.216
B E  1A4YB                 27    0.215  37.578   0.152   0.923   0.002   0.076
E S  1A4YB                 28    0.302  35.348   1.097   0.970   0.001   0.030
B I  1A4YB                 29    0.043   7.973  -0.193   0.970   0.001   0.030
B M  1A4YB                 30    0.086  17.169   0.586   0.970   0.001   0.030
E R  1A4YB                 31    0.446 102.020   0.559   0.923   0.002   0.076
E R  1A4YB                 32    0.340  77.929   0.624   0.858   0.002   0.139
E R  1A4YB                 33    0.317  72.707  -0.587   0.600   0.003   0.397
E G  1A4YB                 34    0.497  39.090  -0.544   0.321   0.003   0.675
B L  1A4YB                 35    0.211  38.652  -0.355   0.257   0.016   0.727
E T  1A4YB                 36    0.441  61.208   0.061   0.181   0.016   0.803
E S  1A4YB                 37    0.604  70.742  -0.014   0.058   0.017   0.925
E P  1A4YB                 38    0.563  79.918   1.355   0.018   0.019   0.964
B C  1A4YB                 39    0.041   5.728  -0.447   0.018   0.019   0.964
E K  1A4YB                 40    0.324  66.606   0.928   0.018   0.019   0.964
E D  1A4YB                 41    0.613  88.405  -0.001   0.018   0.047   0.935
E I  1A4YB                 42    0.413  76.479   0.989   0.053   0.043   0.903
B N  1A4YB                 43    0.179  26.132   0.161   0.018   0.047   0.935
B T  1A4YB                 44    0.081  11.290  -0.148   0.020   0.205   0.775
B F  1A4YB                 45    0.046   9.292   0.469   0.023   0.655   0.322
B I  1A4YB                 46    0.056  10.397   0.415   0.021   0.756   0.223
B H  1A4YB                 47    0.103  18.736   0.470   0.004   0.514   0.481
B G  1A4YB                 48    0.207  16.322  -0.089   0.004   0.138   0.858
E N  1A4YB                 49    0.343  50.142   0.322   0.018   0.047   0.935
B K  1A4YB                 50    0.214  44.102   0.196   0.430   0.016   0.555
E R  1A4YB                 51    0.692 158.445   0.770   0.600   0.003   0.397
E S  1A4YB                 52    0.414  48.509   1.220   0.600   0.003   0.397
B I  1A4YB                 53    0.026   4.884   0.168   0.751   0.050   0.199
E K  1A4YB                 54    0.338  69.629   1.340   0.701   0.107   0.192
E A  1A4YB                 55    0.354  39.022   1.142   0.701   0.107   0.192
B I  1A4YB                 56    0.073  13.450   0.527   0.701   0.107   0.192
B C  1A4YB                 57    0.041   5.756  -0.075   0.502   0.102   0.396
E E  1A4YB                 58    0.574 100.260   0.116   0.181   0.016   0.803
E N  1A4YB                 59    0.401  58.750  -0.539   0.018   0.019   0.964
E K  1A4YB                 60    0.628 129.138  -0.073   0.005   0.015   0.979
E N  1A4YB                 61    0.680  99.508  -0.762   0.005   0.015   0.979
B G  1A4YB                 62    0.140  11.002  -1.588   0.004   0.138   0.858
E N  1A4YB                 63    0.591  86.581   0.699   0.005   0.336   0.660
B P  1A4YB                 64    0.148  20.987  -1.591   0.005   0.262   0.733
E H  1A4YB                 65    0.573 104.247  -0.296   0.004   0.085   0.910
E R  1A4YB                 66    0.661 151.369  -0.124   0.005   0.015   0.979
E E  1A4YB                 67    0.578 101.046  -0.135   0.018   0.019   0.964
E N  1A4YB                 68    0.351  51.460  -0.030   0.019   0.141   0.840
B L  1A4YB                 69    0.092  16.882  -0.896   0.022   0.359   0.619
B R  1A4YB                 70    0.291  66.708  -0.513   0.022   0.552   0.426
E I  1A4YB                 71    0.394  72.982  -0.012   0.021   0.451   0.528
B S  1A4YB                 72    0.162  18.998  -1.183   0.004   0.138   0.858
E K  1A4YB                 73    0.707 145.492  -0.059   0.004   0.085   0.910
E S  1A4YB                 74    0.464  54.357  -0.762   0.005   0.262   0.733
E S  1A4YB                 75    0.451  52.822   1.090   0.004   0.420   0.576
B F  1A4YB                 76    0.064  12.925   0.373   0.004   0.616   0.381
E Q  1A4YB                 77    0.437  78.102   1.432   0.002   0.816   0.182
B V  1A4YB                 78    0.098  15.063   0.254   0.003   0.718   0.279
B T  1A4YB                 79    0.116  16.131   0.291   0.002   0.816   0.182
E T  1A4YB                 80    0.294  40.708   0.462   0.001   0.900   0.099
B C  1A4YB                 81    0.028   3.889   0.340   0.018   0.846   0.136
E K  1A4YB                 82    0.425  87.381   0.749   0.018   0.846   0.136
B L  1A4YB                 83    0.194  35.576   0.021   0.004   0.616   0.381
E H  1A4YB                 84    0.347  63.028   1.128   0.005   0.262   0.733
E G  1A4YB                 85    0.635  49.998  -1.116   0.004   0.085   0.910
E G  1A4YB                 86    0.599  47.165  -1.961   0.005   0.015   0.979
E S  1A4YB                 87    0.596  69.875  -1.067   0.004   0.085   0.910
E P  1A4YB                 88    0.580  82.288   0.092   0.004   0.085   0.910
E W  1A4YB                 89    0.353  84.921   0.015   0.005   0.045   0.951
E P  1A4YB                 90    0.405  57.469  -0.798   0.005   0.045   0.951
E P  1A4YB                 91    0.601  85.225   0.026   0.005   0.045   0.951
B C  1A4YB                 92    0.039   5.433  -1.027   0.019   0.141   0.840
E Q  1A4YB                 93    0.461  82.317   1.202   0.021   0.451   0.528
B Y  1A4YB                 94    0.161  34.363  -0.619   0.022   0.552   0.426
E R  1A4YB                 95    0.463 106.050   0.659   0.022   0.552   0.426
E A  1A4YB                 96    0.391  43.055  -0.956   0.021   0.451   0.528
E T  1A4YB                 97    0.425  58.920   0.378   0.004   0.420   0.576
E A  1A4YB                 98    0.585  64.522  -0.192   0.005   0.262   0.733
E G  1A4YB                 99    0.507  39.862  -0.668   0.004   0.138   0.858
E F  1A4YB                100    0.459  92.161   0.335   0.004   0.197   0.799
E R  1A4YB                101    0.267  61.166   0.974   0.004   0.514   0.481
B N  1A4YB                102    0.188  27.509   1.534   0.001   0.900   0.099
B V  1A4YB                103    0.020   3.120   1.284   0.001   0.959   0.040
B V  1A4YB                104    0.062   9.576   0.921   0.001   0.959   0.040
B V  1A4YB                105    0.017   2.551   1.468   0.001   0.959   0.040
B A  1A4YB                106    0.058   6.425   0.553   0.001   0.900   0.099
B C  1A4YB                107    0.028   3.945   0.408   0.004   0.616   0.381
E E  1A4YB                108    0.409  71.522   0.927   0.004   0.138   0.858
E N  1A4YB                109    0.708 103.637  -1.020   0.005   0.015   0.979
E G  1A4YB                110    0.504  39.665  -0.113   0.005   0.015   0.979
B L  1A4YB                111    0.201  36.766   0.810   0.004   0.197   0.799
B P  1A4YB                112    0.116  16.531   0.127   0.005   0.336   0.660
B V  1A4YB                113    0.051   7.869   0.843   0.021   0.756   0.223
B H  1A4YB                114    0.135  24.520   0.923   0.018   0.846   0.136
B L  1A4YB                115    0.092  16.772   0.636   0.023   0.655   0.322
B D  1A4YB                116    0.246  35.405  -0.073   0.066   0.296   0.638
E Q  1A4YB                117    0.367  65.546   0.097   0.066   0.296   0.638
B S  1A4YB                118    0.202  23.651   0.453   0.022   0.552   0.426
B I  1A4YB                119    0.078  14.485   0.637   0.023   0.655   0.322
B F  1A4YB                120    0.124  24.887  -0.447   0.087   0.683   0.230
E R  1A4YB                121    0.396  90.615  -0.075   0.069   0.386   0.545
E R  1A4YB                122    0.465 106.531   1.032   0.018   0.088   0.893
E P  1A4YB                123    0.781 110.838  -0.638   0.003   0.003   0.994

Explain the output.  Go back.
```

---
